# New Perspectives on the Evolution of Within-Individual Genome Variation and Germline/Soma Distinction

**DOI:** 10.1093/gbe/evab095

**Published:** 2021-05-08

**Authors:** Alexander Suh, Anne-Marie Dion-Côté

**Affiliations:** 1 School of Biological Sciences—Organisms and the Environment, University of East Anglia, Norwich, United Kingdom; 2 Department of Organismal Biology—Systematic Biology, Evolutionary Biology Centre (EBC), Science for Life Laboratory, Uppsala University, Sweden; 3 Département de Biologie, Université de Moncton, New Brunswick, Canada

**Keywords:** DNA elimination, chromatin diminution, germline-restricted chromosome, germline/soma variation

## Abstract

Genomes can vary significantly even within the same individual. The underlying mechanisms are manifold, ranging from somatic mutation and recombination, development-associated ploidy changes and genetic bottlenecks, over to programmed DNA elimination during germline/soma differentiation. In this perspective piece, we briefly review recent developments in the study of within-individual genome variation in eukaryotes and prokaryotes. We highlight a Society for Molecular Biology and Evolution 2020 virtual symposium entitled “Within-individual genome variation and germline/soma distinction” and the present Special Section of the same name in *Genome Biology and Evolution*, together fostering cross-taxon synergies in the field to identify and tackle key open questions in the understanding of within-individual genome variation.

**Significance**

Genome variation within an individual organism can arise through a plethora of mechanisms. Here we provide a perspective on recent developments in the study of within-individual genome variation as highlighted through a virtual symposium and the present Special Section in *Genome Biology and Evolution*, ranging from polyploidy in bacteria, uniparental genome elimination in fishes, mitochondrial heteroplasmy in molluscs, to germline-restricted chromosomes in insects and songbirds. We outline key open questions that can be addressed through combination of diverse methods and diverse study systems.

## Emerging Appreciation of Diverse Forms of Within-Individual Genome Variation

The dynamic nature of organismal genomes is becoming increasingly appreciated. Perhaps the longest known form of within-individual genome variation is somatic mutation, specifically, the movement of transposable elements in maize kernels whose observable phenotype led to the discovery of gene regulation by McClintock (1950, 1956). For the sake of clarity, “germline” refers to the cells or nuclei bearing the genome to be transmitted to the next generation whereas the term “soma” applies to all other cells that may exhibit genome variation relative to each other, or to the germline. Despite these definitions, we emphasize that some organisms do not necessarily have a clear distinction between the germline and soma, and some forms of within-individual genome variation occur in multicellular and unicellular eukaryotes, and even prokaryotes.

**Fig. 1.**
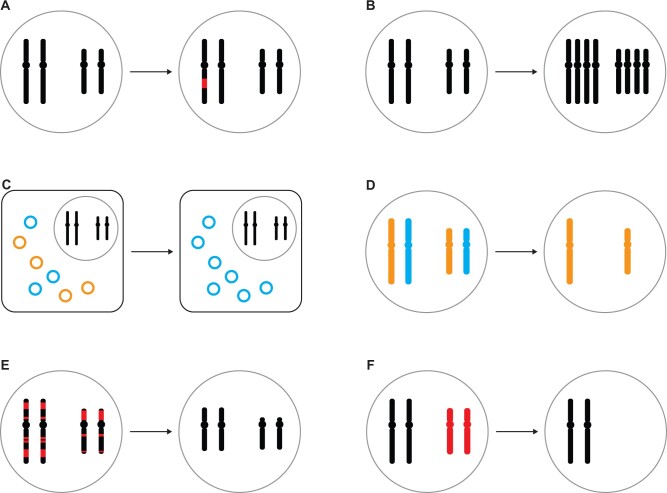
The diversity of within-individual genome variation. The patterns to the left of each arrow reflect the individual’s genome as inherited from the parental generation and to be transmitted to the offspring (“germline”), whereas the patterns to the right of each arrow illustrate genome variation in some cells or nuclei of the individual (“soma”), although further variation may exist within germline and soma, respectively. (*A*) Somatic variation (red) generated by somatic mutation or somatic recombination. (*B*) Somatic variation generated by ploidy change. (*C*) Within-individual mitochondrial heteroplasmy (orange vs. blue). (*D*) Uniparental genome elimination of either maternal or paternal chromosomes (orange vs. blue). (*E*) Programmed DNA elimination of chromosome fragments (red; also known as programmed genome rearrangement or chromatin diminution) from the somatic genome. (*F*) Programmed DNA elimination of entire chromosomes (red; e.g., GRCs) from the somatic genome. Shown are schematic illustrations of a karyotype with metacentric chromosomes inside a nucleus (grey circle), though some of these mechanisms may also apply to holocentric chromosomes of eukaryotes or circular chromosomes of prokaryotes. Note that some of these forms of variation may also arise during meiosis, leading to within-germline genome variation.

Somatic variation may occur through mutations (single-nucleotide changes, small-scale or large-scale structural changes) in individual cells or nuclei during development (fig. 1*A*) and is perhaps best studied in the form of complex mutations in human cancer (Chang et al. 2015; Voronina et al. 2020), retrotransposition in the human brain (Jönsson et al. 2020), and single-nucleotide changes in long-lived plants and fungi (Schmid-Siegert et al. 2017; Hiltunen et al. 2019; Schoen and Schultz 2019). Another type of somatic variation can arise through somatic recombination, such as in the V(D)J locus of human lymphocytes generating genetic variation for antibodies and T-cell receptors (Schatz and Ji 2011). Rather than sequence changes, somatic variation can also arise from ploidy changes during development (fig. 1*B*), with prominent examples being the giant polytene chromosomes in the salivary glands of insects (Stormo and Fox 2017) as well as hepatocytes in mammals (Neiman et al. 2017). Lesser recognized examples are extreme ploidy changes in various groups of unicellular eukaryotes, which contain more than one nucleus (Parfrey et al. 2008), and even some prokaryotes (Angert 2021).

Organellar genomes add another dimension to within-individual genome variation in that different genotypes may coexist (heteroplasmy) and segregate differently during development (fig. 1*C*) (Stewart and Larsson 2014; Breton et al. 2015). Mitochondrial heteroplasmy of some bivalves might be particularly prone to such patterns due to their doubly uniparental inheritance, that is, sex-specific transmission of otherwise coexisting maternal and paternal mitochondria (Zouros et al. 1994; Capt et al. 2020; Stewart et al. 2020), which contrasts sharply with the usually strictly maternal inheritance of animal mitochondria.

Uniparental genome elimination, that is, the elimination of either the maternal or paternal chromosome set during development (fig. 1*D*) (Gardner and Ross 2014), may not necessarily lead to within-individual genome variation if elimination only happens during meiosis. However, in some arthropods with paternal genome elimination such as predatory mites, the paternal chromosomes are not silenced but eliminated from the soma (Nelson-Rees et al. 1980). A form of uniparental genome elimination also exists in some hybrid lineages undergoing hybridogenesis (probably most widely known in *Pelophylax* frogs [Chmielewska et al. 2018]), in which a chromosome complement from one parental species is eliminated without recombination during meiosis (reviewed in Lamatsch and Stöck [2009] and Dalziel et al. [2020]). Fertilization of the haploid oocytes by one of the parental species regenerates diploidy in offspring, which are thus effectively hemiclonal (Lavanchy and Schwander 2019).

An especially peculiar form of within-individual genome variation is caused by programmed DNA elimination during development (fig. 1*E* and *F*). The resulting, often significant, germline/soma genome differences have been observed in a wide range of animals and ciliates (Wang and Davis 2014; Smith et al. 2021), two taxa with an early distinction between germline and soma (germline and somatic cells in animals; micronucleus and macronucleus in ciliates). As a detailed review is beyond the scope of this perspective piece, we point the reader to comprehensive reviews of programmed DNA elimination across ciliates (Chalker and Yao 2011; Bracht et al. 2013; Noto and Mochizuki 2018) and vertebrates (Smith et al. 2021). During programmed genome rearrangement or chromatin diminution (fig. 1*E*), specific regions of chromosomes are eliminated from the differentiating macronucleus in ciliates, as well as from differentiating somatic cells of some nematodes, copepods, lampreys and other animals, leading to extensive genome rearrangements in these organisms (Wang and Davis 2014). Extensive genomic and transcriptomic data in ciliates, nematodes, and lampreys have revealed that eliminated sequences include both germline-expressed genes and repetitive sequences in varying proportions depending on the study system (Smith et al. 2012, 2018; Wang et al. 2012, 2017; Bryant et al. 2016; Hamilton et al. 2016, Timoshevskiy et al. 2019).

Another form of programmed DNA elimination entails the loss of entire chromosomes during germline/soma differentiation (fig. 1*F*), which may either affect sex chromosomes as, for example, in a marsupial species (Close 1984; Wang and Davis 2014) or so-called germline-restricted chromosomes (GRCs) of hagfishes, songbirds, and some arthropods (Wang and Davis 2014; Smith et al. 2021). In lampreys, it was only recently appreciated that not only chromosome fragments but also 12 entire chromosomes are eliminated from somatic cells (Timoshevskiy et al. 2019). Although some insects have numerous GRCs (Hodson and Ross 2021) and the zebra finch GRC is the largest chromosome of its karyotype (Pigozzi and Solari 1998), genomic and transcriptomic data of these GRCs have been restricted to a 19-kb intergenic region of zebra finch GRCs until not so long ago (Itoh et al. 2009). It is only recently that a wealth of sequencing data has provided first glimpses into the sequence content of GRCs of songbirds (Biederman et al. 2018; Kinsella et al. 2019; Torgasheva et al. 2019; Pei et al. 2021) and sciarid flies (Hodson et al. 2021), revealing that GRCs contain many dozens to hundreds of genes and that they may have existed for millions of years in these lineages (Kinsella et al. 2019; Hodson et al. 2021).

Taken together, the study of the diverse forms of within-individual genome variation is currently undergoing a transformation toward more diverse study systems across the tree of life.

## A Society for Molecular Biology and Evolution 2020 Virtual Symposium Showcasing Diversity of the Field

Together with *Genome Biology and Evolution* editor-in-chief Laura A. Katz, we had initially planned a symposium to showcase the diversity of the present topic as part of the Society for Molecular Biology and Evolution (SMBE) 2020 meeting, which was to be held in Québec City on June 28 to July 2, 2020, to foster exchange across study systems and career stages. After pandemic events led to a cancelation of the in-person meeting, we organized the symposium as a free-of-charge virtual event on June 29, 2020. The keynote speaker and the six speakers selected from submitted abstracts for the original in-person meeting all agreed to participate in the virtual symposium. We also solicited additional abstracts for virtual poster presentations on short notice, from which we selected six. Nearly 129 participants registered, representing 35 nationalities working in 24 countries.

The selection of talks and posters spanned the breadth of study systems and career stages among symposium participants. Laurence Hurst gave a 15-min keynote talk entitled “The human early embryo is a selection arena,” and 5-min regular talks from submitted abstracts were given by Esther Angert on “Challenges faced by highly polyploid bacteria with limits on chromosome inheritance,” Marie-Julie Favé on “Multi-omics profiles of somatic mutations in immune cells from an aging human population,” Christina Hodson on “Evolution of a germline restricted chromosome in the fungus gnat *Sciara coprophila*,” Mariangela Iannello on “A naturally heteroplasmic clam shows the effects of genetic bottleneck on paternal mtDNA,” Zuzana Majtanová on “Chromosome dynamics of sexually-parasitic, unisexual carp gudgeons (*Hypseleotris*),” and Jeramiah Smith on “Programmed genome rearrangement in lamprey.” Subsequently, the six poster presenters gave 2-min lightning talks about their posters further highlighting the diversity of study systems, followed by poster presentations in three virtual rooms, which allowed participants to move freely between topics and discussions.

Peak attendance was around 120 participants and our impression was that the real-time virtual symposium with written chat function, combined with a permanent written discussion board, encouraged participants, and especially early-career researchers, to ask questions in a written manner on both platforms, allowing speakers to respond to questions in spoken and written form as time permitted. Taken together, we believe that the free-of-charge virtual format with shorter talks led to participation of researchers from across the world, at all career stages, and may have ultimately increased diversity in this symposium beyond what would have been possible at an in-person symposium.

## A Special Section with New Insights into Within-Individual Genome Variation

In this Special Section of *Genome Biology and Evolution*, we synthesized some of the key insights discussed at the virtual SMBE symposium. Four of the symposium speakers contribute a manuscript with their respective coauthors, and we believe that this selection of manuscripts highlights the diversity of study systems, methods, and concepts for tackling key questions of the field.

Angert (2021) reviews a phenomenon that many eukaryote biologists are probably not aware of—polyploidy in bacteria. Some firmicute bacteria are highly polyploid and produce intracellular offspring instead of binary fission, leading to some chromosome copies effectively having a somatic role by not being passed on to the offspring (Angert 2021).

Majtánová et al. (2021) show that hybrid carp gudgeons undergo uniparental genome elimination, effectively resulting in hybridogenesis. The authors also reveal that genome elimination occurs premeiotically during the juvenile stage, followed by the duplication of the other chromosome complement before meiosis entry (Majtánová et al. 2021). This means that diploid somatic cells bear one copy of each parental species genome, whereas premeiotic germline cells bear two copies of one parental genome.

Iannello et al. (2021) investigate mitochondrial heteroplasmy in a bivalve species with doubly uniparental inheritance. Their results reveal pronounced differences in mitochondrial genotypes among different tissues, possibly as a result of a strong bottleneck early during development (Iannello et al. 2021).

Hodson and Ross (2021) review the diversity of GRCs in dipteran insects, showcasing the known distribution of GRCs among Sciaridae (dark-winged fungus gnats), Cecidomyiidae (gall gnats), and Chironomidae (nonbiting midges). Depending on the taxon, these insects exhibit a single and up to dozens of GRCs with either paternal, maternal, or unbiased inheritance (Hodson and Ross 2021). The authors discuss the potential of genome sequencing for a deeper understanding of GRCs and highlight key questions regarding the evolution of GRCs in dipteran insects.

Finally, Asalone et al. (2021) present an adaptation of a transcriptomics pipeline to detect tissue-specific differences in genome sequencing coverage, caused for example by GRC-linked paralogs of regions derived from regular chromosomes in zebra finch. By aligning genome sequencing reads to a germline genome assembly, their approach detects hundreds of zebra finch germline-restricted contigs based on read depth, 51 of which they validated by quantitative polymerase chain reaction.

## Next Steps toward Elucidating the Evolution of Within-Individual Genome Variation

This Special Section highlights the diversity of within-individual genome variation both in terms of study systems and methods, and that the field is further progressing thanks to the development of cost-efficient or sample-efficient methods for high-throughput data generation. In particular, we anticipate that the continuous improvement of sequencing read length and quality (Sedlazeck et al. 2018) will further increase the resolution for detecting different types of somatic variation, ranging from single-nucleotide variants to large-scale structural variants. Similarly, the development of ultra-low-input libraries for long-read sequencing (Kingan et al. 2019) promises the opportunity of studying within-individual genome variation in organisms with small bodies and/or tissues. However, there is a disconnect between signal/noise in sequencing data and actual chromosome structure, which may remain for some genomic regions until accurate megabase-scale reads are available (Peona et al. 2018), and we therefore emphasize the importance of validating complex genomic results with molecular cytogenetic methods (Deakin et al. 2019).

Which forms of within-individual genome variation are stochastic versus fulfill a biological function remains elusive (box 1), as well as what biological function that might be. The latter is exemplified by the phenomenon of programmed DNA elimination, which has been proposed to either be a means to limit selfish genetic elements to the germline or to minimize antagonistic pleiotropy of genes that are beneficial for the germline but deleterious for the soma (Smith et al. 2012; Wang and Davis 2014; Smith 2017). Comparisons of closely related species are necessary to solve such “chicken or egg” problems, as well as developmental and functional genomics of key candidate genes across different developmental stages. To conclude, the time may have come for agnostic “fishing expeditions” to test whether within-individual genome variation, especially in the form of massive germline/soma genome differences, are the odd exception or the overlooked rule across the tree of life.

**Box 1 Key Questions for the Study of Within-Individual Genome Variation and Germline/Soma Distinction**

1. How common are the different forms of within-individual genome variation across the tree of life?
2. What are the beneficial, neutral, or deleterious effects of the different forms of within-individual genome variation?
3. Are there currently unknown forms of germline/soma, within-soma, or within-germline genome variation that await discovery with new sequencing technologies?

## Data Availability

Not applicable.
